# Improving the Distribution of Rural Health Houses Using Elicitation and GIS in Khuzestan Province (the Southwest of Iran)

**DOI:** 10.15171/ijhpm.2017.101

**Published:** 2017-08-27

**Authors:** Ali Mohammadi, Ali Valinejadi, Sara Sakipour, Morteza Hemmat, Javad Zarei, Hesamedin Askari Majdabadi

**Affiliations:** ^1^Department of Health Information Technology, Paramedical School, Kermanshah University of Medical Sciences, Kermanshah, Iran.; ^2^Social Determinants of Health Research Center, Department of Health Information Technology, School of Allied Medical Sciences, Semnan University of Medical Sciences, Semnan, Iran.; ^3^Health Management and Economics Research Center, Iran University of Medical Sciences, Tehran, Iran.; ^4^Social Determinants of Health Research Center, Saveh University of Medical Sciences, Saveh, Iran.; ^5^Department of Health Information Technology, Paramedical School, Ahvaz Jundishapur University of Medical Sciences, Ahvaz, Iran.; ^6^Nursing Care Research Center, Semnan University of Medical Sciences, Semnan, Iran.

**Keywords:** Rural Health, Geographic Information System (GIS), Iran, Rural Health Services

## Abstract

**Background:** Rural health houses constitute a major provider of some primary health services in the villages of Iran. Given the challenges of providing health services in rural areas, health houses should be established based on the criteria of health network systems (HNSs). The value of these criteria and their precedence over others have not yet been thoroughly investigated. The present study was conducted to propose a model for improving the distribution of rural health houses in HNSs.

**Methods:** The present applied study was conducted in Khuzestan province in the southwest of Iran in 2014-2016. First, the descriptive and spatial data required were collected and entered into ArcGIS after modifications, and the Geodatabase was then created. Based on the criteria of the HNS and according to experts’ opinions, the main criteria and the sub-criteria for an optimal site selection were determined. To determine the criteria’s coefficient of importance (ie, their weight), the main criteria and the sub-criteria were compared in pairs according to experts’ opinions. The results of the pairwise comparisons were entered into Expert Choice and the weight of the main criteria and the sub-criteria were determined using the analytic hierarchy process (AHP). The application layers were then formed in geographic information system (GIS). A model was ultimately proposed in the GIS for the optimal distribution of rural health houses by overlaying the weighting layers and the other layers related to villages and rural health houses.

**Results:** Based on the experts’ opinions, six criteria were determined as the main criteria for an optimal site selection for rural health houses, including welfare infrastructures, population, dispersion, accessibility, corresponding routes, distance to the rural health center and the absence of natural barriers to accessibility. Of the main criteria proposed, the highest weight was given to "population" (0.506). The priorities suggested in the proposed model for establishing rural health houses are presented within five zoning levels –from excellent to very poor.

**Conclusion:** The results of the study showed that the proposed model can help provide a better picture of the distribution of rural health houses. The GIS is recommended to be used as a means of making the HNS more efficient.

## Background

Rural health centers have played a major role in providing primary healthcare and promoting health in rural areas over the last decades in Iran.^1^ The proper geographical distribution of rural health centers, however, is a difficult task filled with many challenges in terms of health equity in developing countries.^2^ First, remoteness, wide geographical dispersion, natural barriers and unsuitable corresponding routes limit the access to health services among rural populations and impede the governments and health policy-makers in planning for equity in health.^3,4^ Second, illiteracy, malnutrition, poverty and poor living conditions make rural populations more prone to diseases than urban populations.^5^ Third, rural areas, especially in developing countries, are less developed,^6-8^ causing an obstructed reception of health services. Fourth, the long physical distance to healthcare centers increases transportation costs for rural dwellers and consequently increases the costs of access to health services for them.^9^ Similar to in other developing countries, in Iran, too, providing health services to rural populations is faced with many of the problems noted.^10^

The need to provide access to health services in villages of Iran was first experienced in the country in the middle of the 1960s, and plans were implemented for distributing health services across villages from the 1970s. After Iran’s Islamic Revolution of 1979, the implementation of health service plans increased in pace as part of the bigger plan for rural development.^11^ In the 1990s, once different plans had been implemented for equity in the distribution of rural health services, the plan for the health network system (HNS) was also executed. The HNS is an integrated system for delivering primary healthcare at four levels: Universities of medical sciences, country health and medical care centers, rural healthcare centers and health houses. The Vice Chancellor for Health at the Ministry of Health and Medical Education is responsible for the policy-making, supervision and regulation of the HNS in the country.^12^ The HNS entails basic principles and rules for the development of healthcare centers.^13^ This plan provides a comprehensive framework for rating and distributing health services with a focus on the “county” as an administrative and geographical measure of the development of health networks. The goals of HNSs include recognizing present and future needs in primary healthcare in the population under coverage, the equitable distribution of healthcare centers, providing easy access to these services, supervising the healthcare services delivered at the first and second levels and referring the patients to higher-level service providers and improving the quality of services.^14^

**Figure 1:**
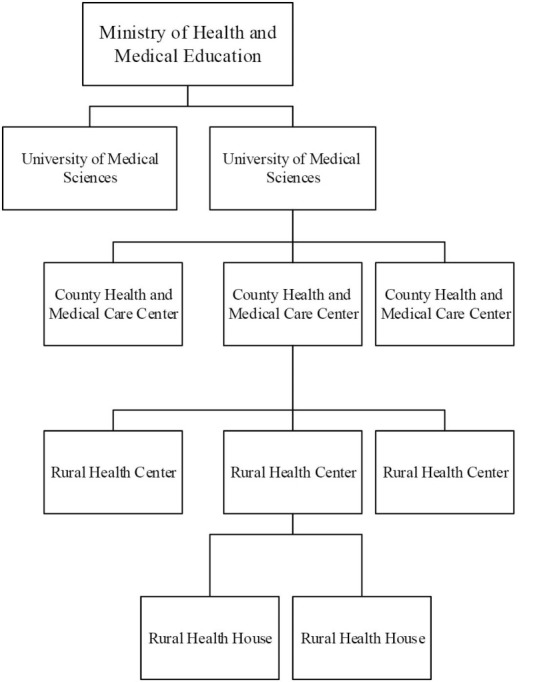
Rural Health Houses in the Health Network System of Iran.

As shown in Figure 1, a rural health house is considered the outermost rural health service provider in the HNS. In the HNS, establishing a rural health house in a village is carried out on the basis of a set of criteria determined by the Health Promotion and Development Office of the Vice Chancellor for Health at the Ministry of Health and Medical Education,^12^ as shown in Table 1. In terms of geographic dispersion, villages should not be more than six kilometers away from the nearest rural health house (ie, one hour’s walking).

**Table 1:** The Criteria Set by the Health Promotion and Development Office of the Vice Chancellor of Health for the Establishment of Rural Health Houses in Iran^14^

**Criteria**	**Attribute**
Geographical location	The units should be located on routine transportation routes. Emphasis on this criterion may sometimes mean that, from the two or more villages that are covered by one unit, one village is selected for the establishment of the designated unit, and this village is not necessarily the most populated one. The presence of one or more of the following factors indicates that a village is on a routine transportation route: ● Being located on the main roadway of neighboring villages. ● Having administrative units like parliament seats, police stations, courts of justice, banks and other governmental institutions. ● Frequent trading, like weekly or regular markets. ● Having schools, high schools, public baths, etc. that can be used by neighboring villages.
The number of population and villages under coverage	Although a consistent pattern cannot be determined for the population covered by each unit according to the regulations and the location of the village, the mean population covered by each unit can be estimated using the calculations related to the activities and duties of each service delivering unit and the staff of the unit: ● About 1500 persons for each health house; however, health houses can be established for more or less populated villages depending on the situation with modifications in their staff and personnel.
Geographic dispersion	If the population is dispersed in different villages, the villages are in the functional domain of a health house if their distance to the health house does not exceed six kilometers (ie, one hour's walking).
Transportation route	The transportation route between the village where the health house is located and the health center should be at least a jeep trail and preferably working year-long.
Distance to the rural health center	The distance between the health house and the health center should preferably not exceed 20 km except for special situations, when a distance of maximum 40 km is acceptable depending on the transportation route and climatic conditions.

The village most adjusted to these criteria is selected as the “main village,” and adjacent villages that are less adjusted to the criteria are then selected as “satellite villages.” Rural health houses are established in the main village, and the dwellers of the satellite villages should visit the main village to receive health services.^12,13^ Rural health houses generally cover a population of 1500 and up to five neighboring satellite villages. Each rural health house is staffed with rural health workers called “*Behvarz.*”^15^

In Iran, rural health houses have a variety of responsibilities, including public health training, prenatal healthcare, child care (for ages 0-8 years), family planning, immunization, environmental sanitation and certain primary health services (such as dressing wounds, injections, etc).^14^ Studies reveal the effectiveness of health houses in family planning policies, reduced population growth, improved mother and child health and improved health indicators in the rural population of the country.^11,16,17^

Given the role of rural health houses in rural health programs in the country, managing the optimal distribution of rural health houses is crucial. However, the efficient management of rural health services requires comprehensive and accurate information about population distribution, geographical factors, rural infrastructures, corresponding routes’ conditions, etc. Making decisions about this issue thus requires the integration of different data and a comprehensive analysis of all the influential factors. One of the most efficient tools for the management of the optimal accessibility and distribution of health services in rural areas is the geographical information system, abbreviated as GIS.^18^ The main advantage of the GIS is its potential for the simultaneous use of descriptive and spatial data, which allows complicated analyses on maps and a variety of modelling to be performed using combined descriptive and spatial data.^19^ The GIS and its relevant method of spatial analysis provide a set of tools for interpreting the geographical distribution of health services across the country and contribute to evidence-based decision-making.^20^ An efficient and optimal site selection in the GIS depends on providing accurate definitions for the criteria, which is crucial to decision-making and prioritization. There are different methods for the prioritization of sets of criteria. One of the most common methods used is the analytical hierarchy process, abbreviated as analytic hierarchy process (AHP). The AHP is a structured quantitative method that contributes to the selection of an option from the various solutions that exist to a problem.^21^ The integration of the AHP and the GIS is an efficient approach to site selection.^22^ The particular conditions of Khuzestan province make the appropriate distribution of rural health houses in this province a task that requires a more efficient management. The first reason is that Khuzestan is considered the hub of agriculture in Iran.^23^ The health of the rural population in this area is therefore particularly important. The second reason is that Khuzestan boasts a higher ethnical, geographical, economic and social diversity in its rural areas compared to the other provinces of the country.^24^

The third reason is that the region’s extremely warm and humid weather during some seasons of the year^25^ and the continuous dust storms in the province limit the rural population’s access to health houses. Given the importance of health houses for rural dwellers’ health and the challenges that exist in the proper management of their distribution according to the HSN, the present study was conducted to present a model for the optimal distribution of rural health houses using the AHP in the GIS environment.

The results of this study may provide an objective and accurate model for the distribution of rural health houses and thus help health network managers and other health decision-makers in the distribution and management of rural health houses. Moreover, this study may provide a suitable framework for conducting similar studies in other areas and on other health services.

## Methods

The present applied study was conducted with a descriptive analytical approach in 2014-2016. The study population consisted of the villages and rural health houses of Khuzestan province in 2014. All the villages and rural health houses of the province were examined as the statistical population of the study without any sampling. This study was conducted within ten steps.

### Basic Data Collection

This step was dedicated to collecting the required descriptive and spatial data. The descriptive data pertaining to the villages including the name of county, the name of the village, population, number of households, residential status of the village (permanent, seasonal, deserted), type of transportation route, basic developmental infrastructures such as water, electricity, gas, telephone, and the presence of educational institutions (kindergarten, primary school, etc), administrative and governmental units (police station, the county seat, city council), healthcare facilities (rural health houses, rural healthcare center, pharmacy, doctor’s office), business and market places (local market, gas/petrol station, garage, restaurant). The descriptive data pertaining to the rural health houses including the name of the rural health house and its corresponding rural healthcare center, the village type (main or satellite) and population under coverage by the health houses.

The spatial data of Khuzestan province, including the longitude and latitude of the village, topographic layers and the counties, villages and rural roads (scale: 1:25000) according to the latest administrative divisions of the country.

The data collection tool for gathering the descriptive data was a checklist that was designed according to the objectives of the study and after consultation with HSN and GIS experts. The validity of the checklist was confirmed by five public health experts with an experience of working in the HSN and three senior GIS experts. To access the descriptive data pertaining to the villages, the researchers visited the Statistical Center of Iran. The required spatial data were then collected by visiting the National Cartographic Center and Khuzestan Province Governor Generalship.

### Completion and Correction of the Collected Data

The primary analysis of the data collected from the Statistical Center of Iran showed that some of the data pertaining to villages with a population less than five families had not been properly recorded. Moreover, there were contradictions between the data collected from the Statistical Center of Iran and the data collected from Ahvaz and Dezful universities of medical sciences regarding the name of the villages. The researchers therefore visited Khuzestan province governor generalship to complete the data and correct the contradictions.

### Create Geographic Information System Geodatabase

Descriptive and spatial data were entered into the GIS software and a database was created using the ArcGIS 9.3 software.

### Defining the Main Criteria for an Optimal Site Selection

This step entailed defining the main criteria for an optimal site selection in the distribution of rural health houses according to the HNS criteria. A list of the main criteria and the sub-criteria was first prepared in compliance with the HNS criteria and through consulting with HNS experts, health network managers and GIS experts. The list included seven main criteria along with their definitions, and was then distributed among the experts in the form of a questionnaire that consisted of eight closed-ended questions and one open-ended question. A 5-point Likert scale (4 for very high priority and 0 for not a priority) was used to evaluate the answers given to the questions. The content validity of the questionnaire was confirmed using the opinion of five experts (three HNS experts and two GIS experts). These experts were excluded from the reliability confirmation step of the study.

The test-retest method was used to confirm the reliability of the questionnaire, which was distributed among seven experts, including three HNS experts, three urban health network deputies and one GIS expert. Two weeks later, the questionnaire was distributed once again among the same seven experts. The Pearson correlation coefficient was calculated as 0.84, confirming the reliability of the questionnaire. It should be noted that these experts were excluded from the next step (ie, surveying the experts).

### Confirming the Main Criteria for an Optimal Site Selection

The criteria defined in the previous step were confirmed using the experts’ opinions. A total of 20 experts were selected based on their relevant experience and knowledge and through purposive sampling, 10 of whom were experts in HNS, four were former health network managers in counties of Khuzestan province, four were GIS experts with experience in site selection projects in rural areas and two were health workers (ie, *Behvarz*). To collect the experts’ opinions, the questionnaire was distributed among them in person or through email. The items that received a mean score of 2 out of 4 were ultimately selected as the main criteria for site selection and the items receiving a score lower than 2 were excluded from the list of the criteria.

### Defining the Sub-criteria

This step involved defining sub-criteria for the criteria confirmed in the previous step, for which a meeting was held with two HNS experts and one GIS expert. Five sub-criteria were defined for each main criterion.

### Weighting the Main Criteria and the Sub-criteria

The seventh step involved weighting the main criteria and the sub-criteria using the AHP. To determine the coefficient of importance (weight), the main criteria and the sub-criteria were compared in pairs, and the coefficients of importance obtained were then entered into a matrix of pairwise comparison of the criteria. The pairwise comparisons were performed using Saaty’s 9-point scale (Table 2). The experts’ opinions also aided the comparison of the criteria. To conduct the pairwise comparison of the main criteria and the sub-criteria, a meeting was held with three HNS experts and one GIS expert. The results were then entered into Expert Choice-9, and the relative, normal and final weight and the inconsistency rate of the main criteria and the sub-criteria were thus calculated. Another meeting was held with the experts to revise some of the scores given to the sub-criteria that had a high inconsistency rate. After the revisions, the total inconsistency rate for the main criteria was calculated as 0.08, which is considered an acceptable rate.

**Table 2:** The Saaty Rating Scale for Pair-Wise Comparisons^26^

**Intensity of Importance**	**Definition**	**Explanation**
1	Equal importance	Two factors contribute equally to the objective.
3	Moderate importance	Experience and judgment slightly favor one over the other.
5	Strong importance	Experience and judgment strongly favor one over the other.
7	Very strong importance	Experience and judgment very strongly favor one over the other. Its importance is demonstrated in practice.
9	Extreme importance	The evidence favoring one over the other is of the highest possible validity.
2,4,6,8	For compromise between above values	When compromise is needed.

### Forming Layers for the Main Criteria and the Sub-criteria Weighted in ArcGIS

A layer was prepared for each of the main criteria and the sub-criteria in ArcGIS. The layers formed through the sub-criteria were then combined based on their relative weight, and a common layer was formed for each criterion. A comprehensive map (overlaying all the layers) was created through combining the layers formed for the criteria and the ones formed for the villages (including layers for village dispersion, corresponding routes, rural infrastructures and topography).

### Proposing an Optimal Model for the Distribution of Rural Health Houses in the HNS

The optimal model for the distribution of rural health houses was proposed based on the final map and in compliance with the HNS criteria. The model suggested an optimal site selection for establishing rural health houses within five zoning levels (from excellent to very poor).

### Examining the Current Distribution of Rural Heath Houses in the Proposed Model

In this step, layers were formed in ArcGIS for the data pertaining to the health houses, including layers for the distribution of health houses, the population covered by the health houses, the span of services and the satellite villages covered by each health house. These layers were then integrated with the comprehensive map of the proposed model and the distribution status of the health houses was determined according to this model.

## Results

### Population and Residential Status in the Villages of Khuzestan Province

**Figure 2:**
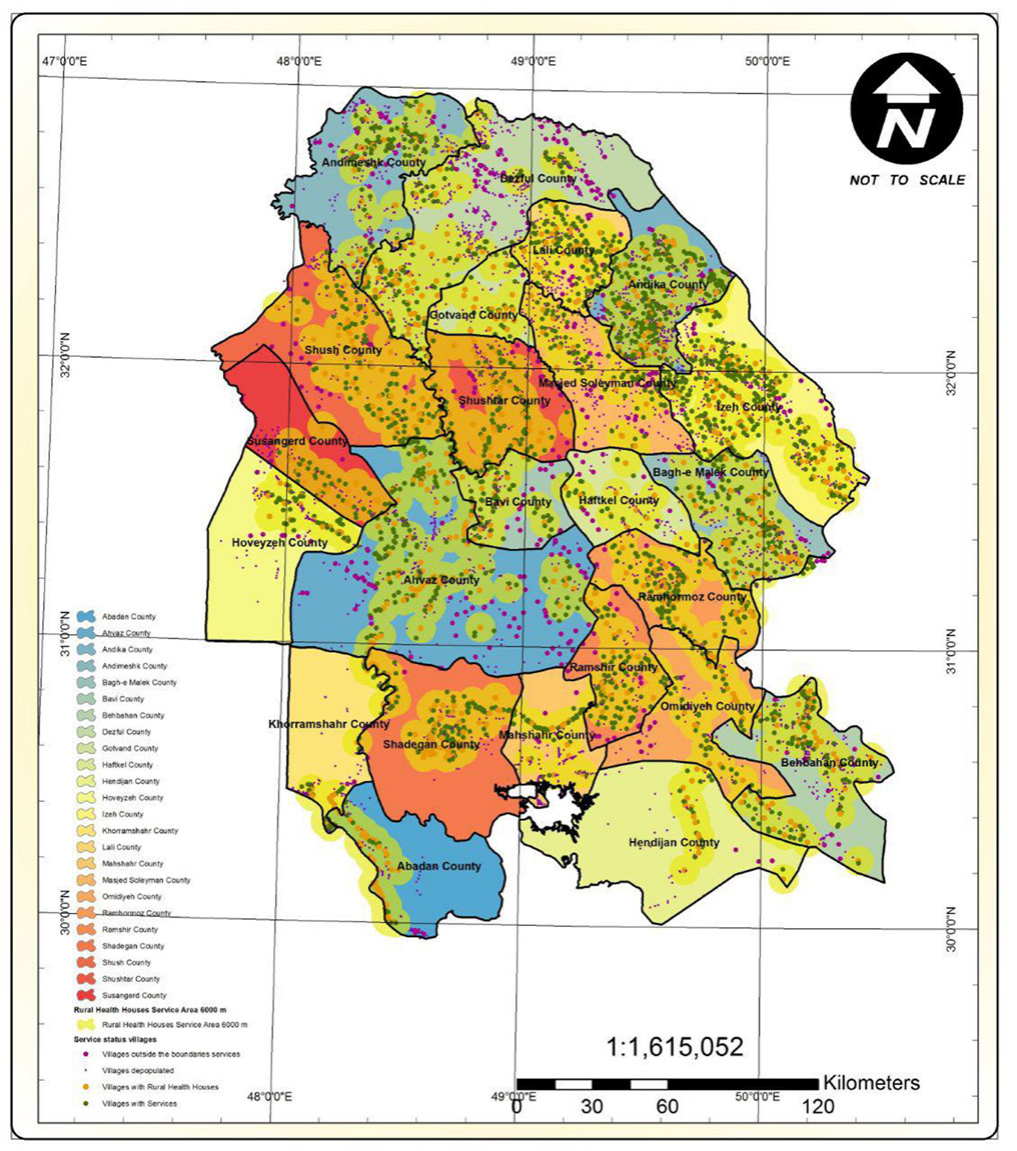
The Geographic Distribution of Villages in Khuzestan Province.

Khuzestan province has a population of 4 531 720 within 1 083 341 families, with 290 052 families living in rural areas (26.8%). Khuzestan has 4547 villages, 3461 (76.1%) of which were inhabited at the time the study was conducted. Regarding residential status, 3315 villages (72.9%) had permanent residents, 146 villages (3.2%) had seasonal residents, and 985 villages (23.9%) were uninhabited (Figure 2). The mean population of the villages was 282. Villages with a population less than 250 were the most prevalent (41.4%) among the inhabited villages.

### The Geographical Dispersion and Service Span of Rural Health Houses

**Figure 3:**
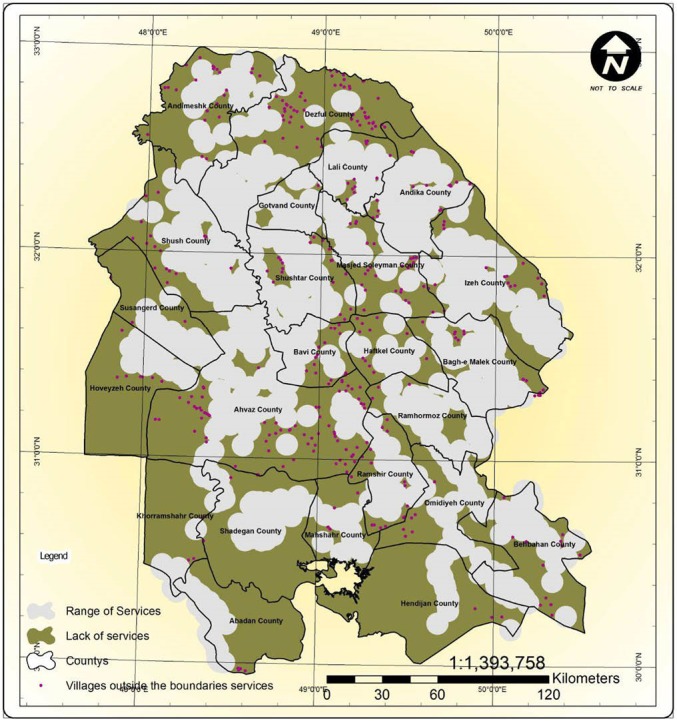
The Inhabited Villages Outside the Standard Service Span of the Rural Health Houses (a 6-km Radius).

There were 896 health houses in Khuzestan. The service span of the health houses showed that 358 of the inhabited villages were over 6 km away from the nearest health house and were thus located outside the standard service span of the health houses (Figure 3).

### The Main Criteria for an Optimal Site Selection for Rural Health Houses From the Experts’ perspectives

Of the total of seven criteria proposed for an optimal site selection for rural health houses, the experts chose six as the main criteria, including developmental and welfare infrastructures, population, geographical dispersion (ie, the distance of the villages to rural health house), transportation routes, distance to the rural health center and the absence of natural and artificial barriers to accessibility (Table 3).

**Table 3:** The Main Criteria for an Optimal Site Selection for Rural Health Houses From the Experts’ Perspective

The Main Criteria	Welfare and Developmental Infrastructures	Population	Geographical Dispersion	Distance to the Rural Health Center	Absence Of Natural and Artificial Barriers to Accessibility	Transportation Routes	Absence of Ethnic and Cultural Conflict
Mean (score of 4)	3.35	3.80	3.30	3.15	3.60	3.40	1
Result	Accepted	Accepted	Accepted	Accepted	Accepted	Accepted	Not accepted

### The final, normal and relative weight of the main criteria and the sub-criteria for an optimal site selection for rural health houses (based on the AHP)

The results of the AHP performed on the main criteria for an optimal site selection for health houses showed the highest priority coefficient to pertain to the “population” criterion with a relative weight of 0.506, and the lowest to pertain to the “distribution” and “distance to the rural health center” criteria with relative weights of 0.037. The results of the AHP performed on the sub-criteria showed the highest priority coefficient to pertain to “a population over 1000” with a final weight of 0.192, and the lowest to pertain to “a 5 to 6 km distance” with a final weight of 0.0013 (Table 4).

**Table 4:** The Final, Normal and Relative Weight of the Main Criteria and the Sub-criteria for an Optimal Site Selection for Rural Health Houses

**The Main Criteria**	**Sub-criteria**	**The results of the AHP**			
		**Relative Weight of the Main Criteria**	**Relative Weight of Sub-criteria**	**Normal Weight of Sub-criteria**	**Final Weight of Sub-criteria**
Welfare and developmental infrastructures	Having healthcare facilities	0.140	0.287	0.040	0.0403
	Having administrative and governmental unites		0.108	0.015	0.0152
	Having business and market places		0.143	0.020	0.0201
	Having basic developmental infrastructures (water, electricity, gas, telephone)		0.217	0.030	0.0304
	Having educational institutions		0.245	0.034	0.0344
Population	Population less than 250 individuals	0.506	0.029	0.015	0.0147
	Population 250 to 500 individuals		0.081	0.041	0.0411
	Population 500 to 750 individuals		0.189	0.096	0.0958
	Population 750 to 1000 individuals		0.323	0.163	0.1638
	Population more than 1000 individuals		0.379	0.192	0.1922
Geographical dispersion	Less than 2 km distance to RHS	0.037	0.537	0.020	0.0199
	1 to 2 km distance to RHS		0.248	0.009	0.0092
	3 to 4 km distance to RHS		0.124	0.005	0.0046
	4 to 5 km distance to RHS		0.055	0.002	0.0020
	5 to 6 km distance to RHS		0.036	0.001	0.0013
Transportation routes	Paved road	0.075	0.605	0.045	0.0455
	Gravel road		0.179	0.013	0.0135
	Dirt road		0.082	0.006	0.0062
	Jeep trail road		0.052	0.004	0.0039
	Railroad		0.082	0.006	0.0062
Distance to the RHC	Less than 5 km distance to RHC	0.037	0.576	0.021	0.0214
	5 to 10 km distance from RHC		0.216	0.008	0.0080
	10 to 15 km distance from RHC		0.118	0.004	0.0044
	15 to 20 km distance to RHC		0.056	0.002	0.0021
	More than 20 km distance to RHC		0.034	0.001	0.0013
Absence of natural and artificial barriers to accessibility	Elevation barriers (mountains, hills, valleys, etc)	0.204	0.183	0.037	0.0374
	Water barriers (rivers, lakes, wetlands, etc)		0.341	0.070	0.0697
	Vegetation barriers (forest, bush, farm, etc)		0.065	0.013	0.0133
	Desert barriers (sabulous salt marshes, etc)		0.042	0.009	0.0086
	Other artificial barriers (minefield, military exclusion zone, etc)		0.369	0.075	0.0754

Abbreviations: AHP, analytic hierarchy process; RHC, rural health center.

### The Optimal Site Selection Model for Health Houses

**Figure 4:**
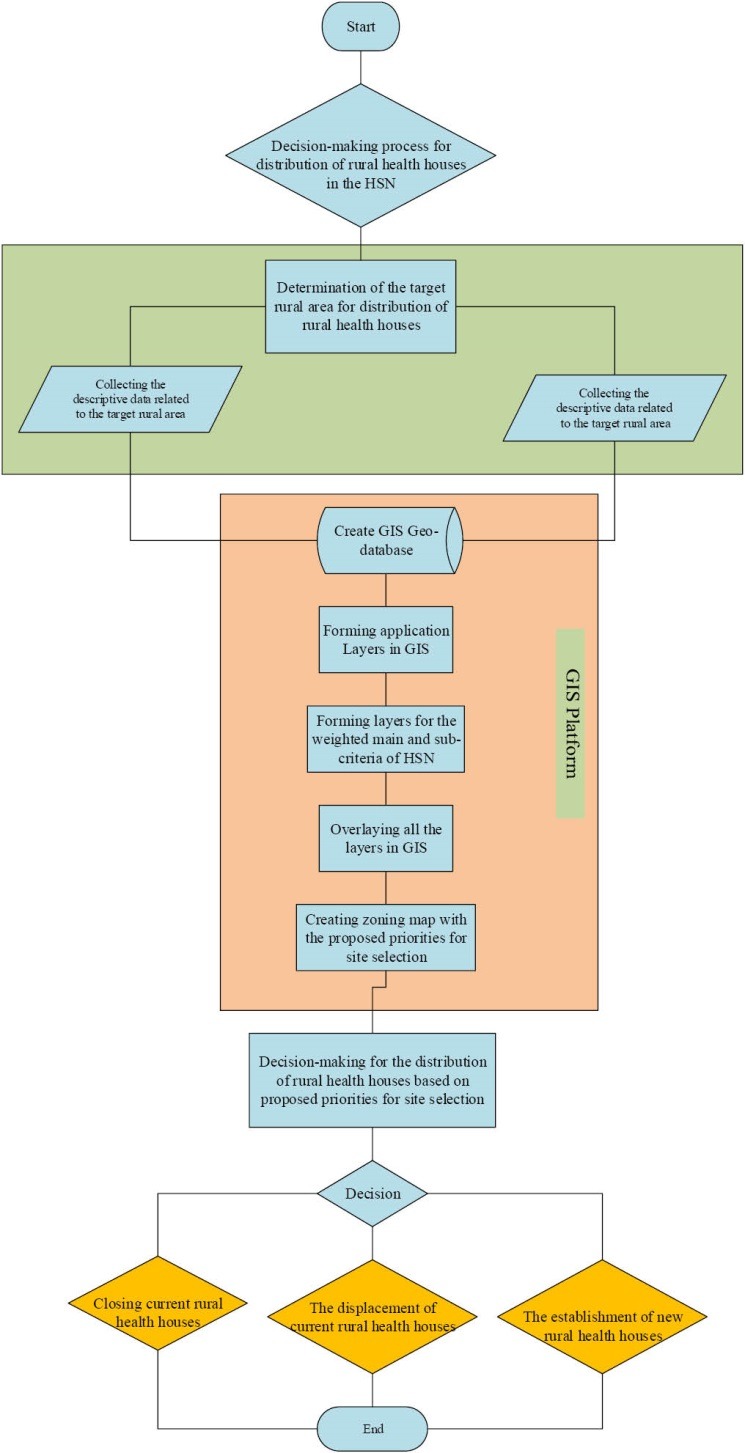
The Model Proposing Optimal Sites Selection for Establishing Rural Health Houses.

The optimal model for the distribution of health houses according to the HNS suggests optimal sites for establishing health houses on the basis of the GIS. In this model, the proposed sites for establishing rural health houses were presented within five zoning levels, from excellent to very poor. Figure 4 shows the model for the distribution of rural health houses in a target rural area.

### The distribution Status of Rural Health Houses in Khuzestan Province in the Proposed Model

**Figure 5:**
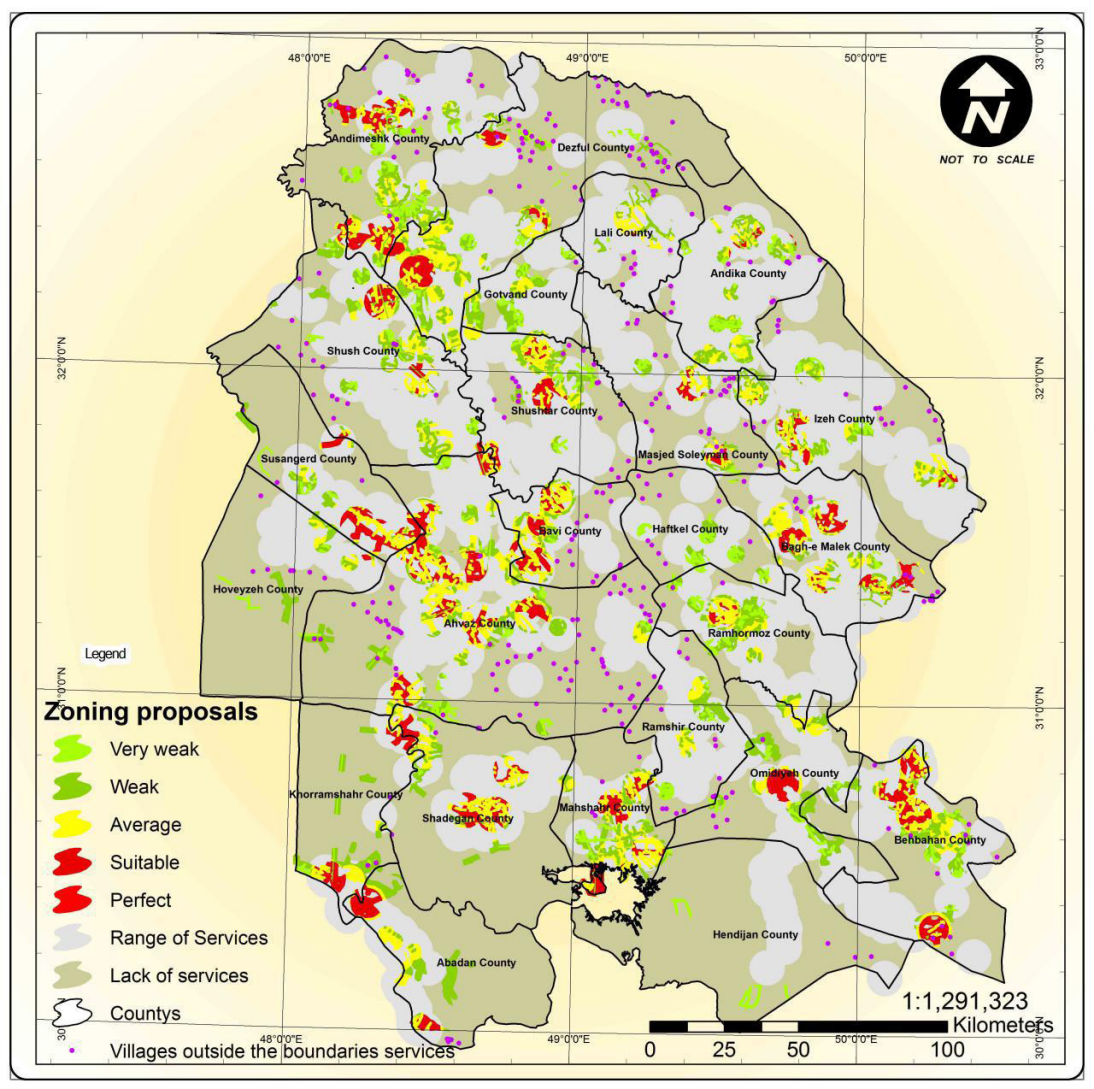
The Distribution Status of Rural Health Houses in Khuzestan Province in the Zoning Levels Proposed for an Optimal Site Selection for Establishing Health Houses.

According to the proposed model, of the total of 896 health houses, 185 were located in an excellent zone, 376 were in a good zone, 210 in a moderate zone, 123 in a poor zone and 2 in a very poor zone (Figure 5).

## Discussion

The present study determined “population,” “welfare and developmental infrastructures,” “dispersion,” “Transportation routes,” “distance to the rural health center,” and “the absence of natural and artificial barriers to accessibility” as the main criteria for an optimal site selection from the perspective the experts. In a study conducted to determine the optimal spatial pattern of rural service centers in northern Khuzestan, Mekaniki and Sadeghi proposed population density, distance from the roads, altitude, land slope and developed infrastructures as the main criteria.^27^ and Møller-Jensen determined a set of criteria and proposed a framework for providing primary health services to rural areas in Ghana, consisting of population, proximity, developed infrastructures and centrality.^28^

Of the main criteria proposed in the present study for an optimal site selection, “population” and “the absence of natural and artificial barriers to accessibility” were found to be the most important according to the experts. In another study conducted in Iran to prioritize the factors affecting site selection for rural health centers with “telehealth services” implemented, “population” and “accessibility” were found to have the highest priority among all the criteria defined.^29^ One of the objectives of site selection for health centers is to enable the proper providing of services to the largest possible population. The location of health centers gains more importance as the population increases, and loses its significance with the decline of the population. In rural areas, in addition to distance and corresponding routes, the type of land and its tolls affect the time spent to access the health center. The presence of natural and artificial barriers can limit physical access to the health center for rural dwellers to a great degree.^30^ The proposed model used all the main criteria and the sub-criteria determined in zoning optimal sites for the distribution of health houses. The sites proposed for establishing health houses were classified into five zones, from excellent to very poor. The excellent sites were villages with a large population, paved roads and a short distance to the rural health center and boasting the highest number of welfare and developmental infrastructures and lacking any natural and artificial barriers to accessibility. The proposed model’s analysis of the distribution of health houses in Khuzestan province showed that a number of health houses were located in unsuitable zones, although most of them were in fact located in suitable zones. The sites of these health houses were unsuitable due to factors such as having a population less than 500, being located at a distance over 20 km from the main village with the relevant health center, having unsuitable corresponding routes, better conditions in a satellite village than in the main village (ie, being better adjusted to the HNS criteria), the presence of natural barriers to accessibility in the satellite villages, and a distance over 6 km from the satellite villages to the health house located in the main village.

## Conclusion

The proposed model is consistent with the HNS criteria, has been designed based on the opinions of experts and uses AHP for the analysis of the spatial and descriptive data and can thus help HNS experts better understand the distribution status of health houses and facilitate an evidence-based decision-making system for the distribution of rural health services. Given the complicated nature of the subject of the distribution of health services in rural areas and the influence of geographical parameters in decision-making, GIS is recommended to be used for the better planning and management of HNS in rural areas. For this purpose, the principles of surveying and mapping, telemetry and using the GIS can be taught to HNS users. A telemetry and GIS unit can also be established in the health deputies of medical universities across the country. Telemetry and GIS experts can then be employed to make the work performed in these units more efficient.

## Acknowledgements

This study was supported by Iran University of Medical Sciences, Tehran, Iran (grant No: 17005). The authors would like to thank all of the participants.

## Ethical issues

Ethical issues such as plagiarism, conflict of interest, data fabrication, data falsification, privacy informed consent, misconduct, double publication and/or submission, redundancy, etc have been completely observed by the authors. Ethics committee of Iran University of Medical Sciences, Tehran, Iran approved the study with ethical approval code: IR.IUMS.REC 1391.17005.

## Competing interests

Authors declare that they have no competing interests.

## Authors’ contributions

AM: writing the first draft and contribution to final draft, collection of data and analysis. JZ: supervision of group, design of study, contribution to first draft, final draft and collection of data and analysis. AV: contribution to first draft, final draft and collection of data and analysis. SS: collection of data. MH: collection of data. HAM: collection of data and contribution to final draft.

## Authors’ affiliations

^1^Department of Health Information Technology, Paramedical School, Kermanshah University of Medical Sciences, Kermanshah, Iran. ^2^Social Determinants of Health Research Center, Department of Health Information Technology, School of Allied Medical Sciences, Semnan University of Medical Sciences, Semnan, Iran. ^3^Health Management and Economics Research Center, Iran University of Medical ‎Sciences, Tehran, Iran. ^4^Social Determinants of Health Research Center, Saveh University of Medical Sciences, Saveh, Iran. ^5^Department of Health ‎Information Technology, Paramedical School, ‎Ahvaz Jundishapur University of Medical Sciences, Ahvaz, Iran. ^6^Nursing Care Research Center, Semnan University of Medical Sciences, Semnan, Iran.

## Key messages

**Implications for policy makers**

- As a simple case of using geographic information system (GIS) in rural health management, the present study showed that GIS offers a variety of opportunities and tools to help regional and national health policy-makers use geography as a framework for better addressing problems and evaluating the proposed solutions implemented in a comprehensive, analytic and visual manner.
- The distribution of rural health centers, such as rural health houses, depends on many criteria, and the more accurate and quantitative are those criteria, the more reliable and valid will be the decisions made.
- GIS is recommended when policy-makers are faced with a large rural area like Khuzestan Province, which is very different in terms of developmental infrastructures and geographic characteristics.

**Implications for public**

The rural health houses of Khuzestan province, just like those in other provinces of Iran, play an important role in providing primary healthcare. Nonetheless, suitable and fair access to rural health houses is difficult for healthcare systems. Rural communities in Iran have been faced with rapid changes over the past years. Youth migration to big cities, villages becoming more and more isolated, the improvement of rural infrastructures, the growing costs of healthcare and the changes in the priorities of primary healthcare programs require innovations to improve the distribution of rural health houses. This study can serve as evidence for the innovations made in planning for the distribution of rural health centers.
